# Cutaneous Leukocytoclastic Vasculitis as a rare adverse event of infliximab in Crohn’s disease: a case report

**DOI:** 10.1093/omcr/omag067

**Published:** 2026-05-10

**Authors:** Houssam EL Mourad, Esraa Alsayed, Tamadar AL Doheyan, Yasser Alomar, Othman Alharbi

**Affiliations:** Gastroenterology & Hepatology Department, King Saud University, alyasmmen, street 140, home3-4 Riyadh, Saudi Arabia; Gastroenterology & Hepatology Department, King Saud University, alyasmmen, street 140, home3-4 Riyadh, Saudi Arabia; Pathology Department, King Saud University, Riyadh, Saudi Arabia; Gastroenterology & Hepatology Department, King Saud University, alyasmmen, street 140, home3-4 Riyadh, Saudi Arabia; Gastroenterology & Hepatology Department, King Saud University, alyasmmen, street 140, home3-4 Riyadh, Saudi Arabia

**Keywords:** Crohn’s disease, infliximab, leukocytoclastic vasculitis, anti-TNF, adverse event

## Abstract

Leukocytoclastic vasculitis (LCV) is a rare, immune-mediated small-vessel vasculitis that can be triggered by infections, autoimmune diseases, or medications, including biologic therapies. We present a case of a patient with Crohn’s disease who developed biopsy proven LCV during longterm infliximab therapy. After four years of sustained remission on infliximab, she developed cutaneous palpable purpura affecting the lower extremities. Laboratory evaluation excluded systemic vasculitis, infection, and other secondary causes. Histopathology confirmed leukocytoclastic vasculitis. Discontinuation of infliximab, led to complete resolution of skin lesions. This case underscores the importance of clinician awareness of rare biologic-associated adverse effects and highlights the need for timely recognition and management to prevent systemic involvement.

## Introduction

Crohn’s disease (CD) is a chronic inflammatory bowel disease characterized by transmural gastrointestinal inflammation. Biological therapies targeting tumor necrosis factor-alpha (TNFα), such as infliximab, have revolutionized the management of moderate to severe CD by inducing and maintaining remission [1]. Despite their efficacy, anti-TNF agents are associated with a spectrum of adverse events, ranging from infusion reactions and infections to immune mediated phenomena, including paradoxical autoimmune disorders [2].

Leukocytoclastic vasculitis (LCV) is an uncommon small-vessel vasculitis characterized histologically by neutrophilic infiltration, leukocytoclasia (fragmentation of neutrophil nuclei), and variable fibrinoid necrosis of vessel walls [3]. Anti-TNF–associated leukocytoclastic vasculitis is rare. In a multicenter retrospective cohort of 2442 IBD patients, five cases of LCV were identified among 862 patients exposed to anti-TNF therapy (≈0.6%), most occurring in patients treated with adalimumab [4]. These data underscore the rarity of this adverse event.

Clinically, it typically manifests as palpable purpura, most often involving the lower extremities. Although LCV may occur as an extraintestinal manifestation of inflammatory bowel disease, drug-induced vasculitis must be carefully considered, particularly in patients receiving biologic therapies [5, 6].

Here, we describe a patient with Crohn’s disease who developed infliximab-associated leukocytoclastic vasculitis, emphasizing the need for early recognition and appropriate management.

## Case presentation

A 52-year-old woman with a history of fistulizing ileocolonic Crohn’s disease with perianal involvement underwent total colectomy 16 years prior to presentation.

She had been treated with 5-aminosalicylates and intermittent courses of corticosteroids for many years. Due to ongoing clinically and endoscopically active disease, infliximab was initiated at a dose of 5 mg/kg at weeks 0, 2, and 6, followed by maintenance therapy every 8 weeks.

Following initiation of infliximab, the patient achieved sustained clinical and biochemical remission, with resolution of diarrhea, normalization of inflammatory markers, fecal calprotectin levels below 20 μg/g, and endoscopic remission (Rutgeerts score i0).

After four years of continuous infliximab therapy, she developed non-blanching, tender, erythematous lesions over both lower extremities. The eruption initially appeared as small pruritic pinpoint lesions and was treated with topical corticosteroids with partial improvement. Over time, the rash progressed in extent and tenderness, involving both legs, arms, and forearms, despite continued infliximab therapy.

At the time of rash onset, she reported no fever, weight loss, urinary symptoms, hematuria, or gastrointestinal complaints. Importantly, no new medications, including antibiotics or nonsteroidal anti-inflammatory drugs, had been introduced prior to symptom onset.

**Figure 1:**
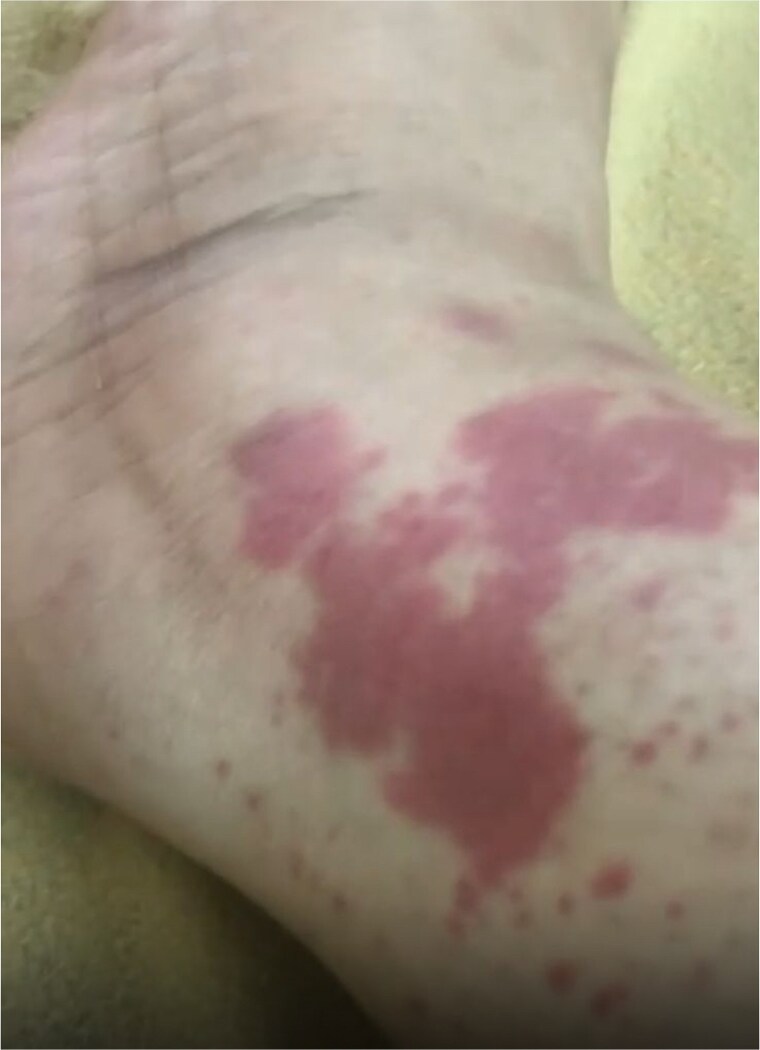
Palpable purpuric macules and papules involving the lower extremities, non-blanching on pressure.

**Figure 2:**
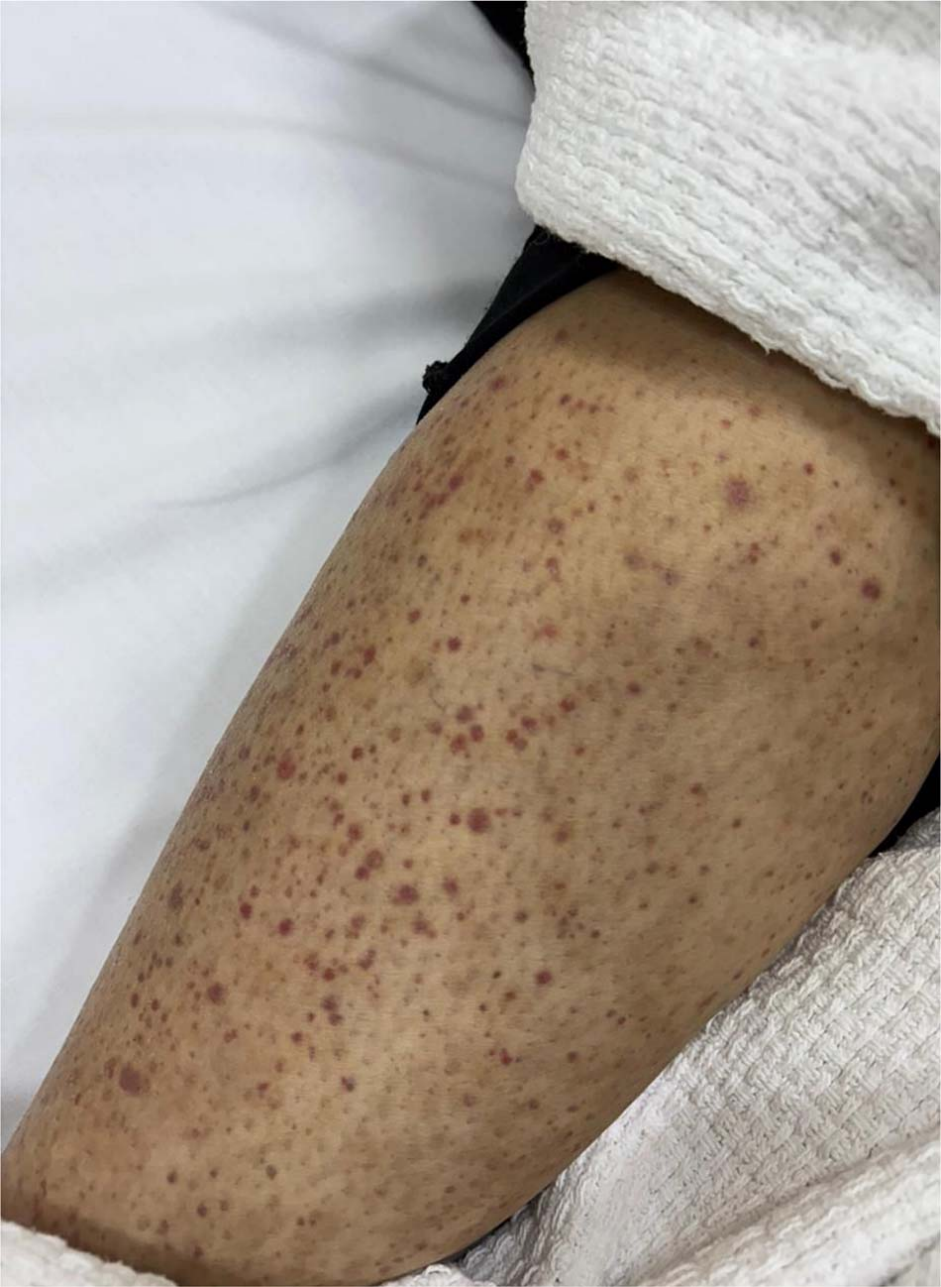
Extension of purpuric lesions to the lower legs and ankles.

**Figure 3:**
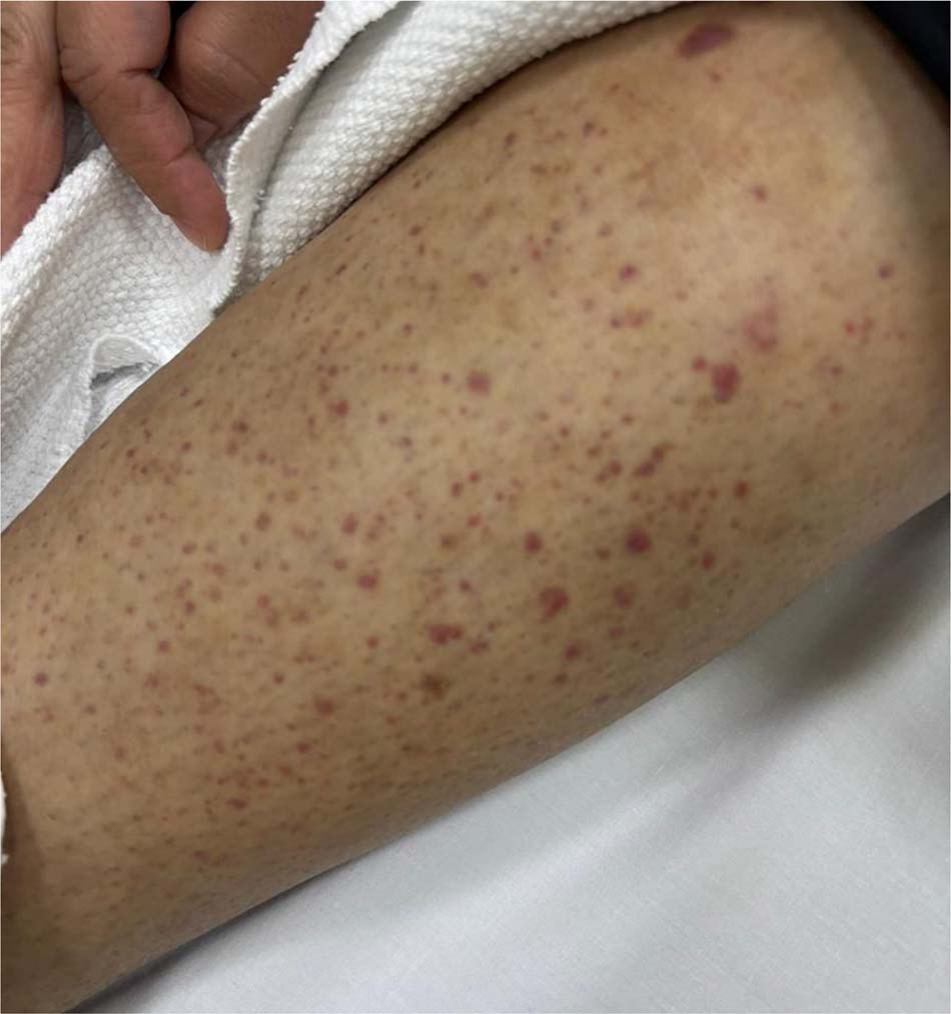
Confluent erythematous-purpuric plaques involving lower extremity.

On examination, she was vitally stable. Pulmonary auscultation was clear bilaterally, and cardiac examination revealed normal S1 and S2 with no murmurs. Abdomen soft and lax without tenderness. Redish-Brownish Multiple, discrete to coalescent palpable purpuric macules and papules, some forming larger confluent patches. Lesions range from pinpoint petechiae to several centimeter-sized plaques. Non-blanching on pressure, No ulceration, vesiculation, or necrosis observed (Figs 1–3).

Laboratory investigations demonstrated normal hemoglobin, albumin, and platelet counts. Coagulation studies, including prothrombin time, international normalized ratio, and activated partial thromboplastin time, were within normal limits. Fecal calprotectin remained low. Viral serologies for hepatitis A, B, and C, herpes simplex virus, and cytomegalovirus were negative.

Autoimmune testing, including antinuclear antibodies and antineutrophil cytoplasmic antibodies, was negative. Infliximab trough level was therapeutic (12 μg/ml) with no detectable anti-drug antibodies.

**Figure 4:**
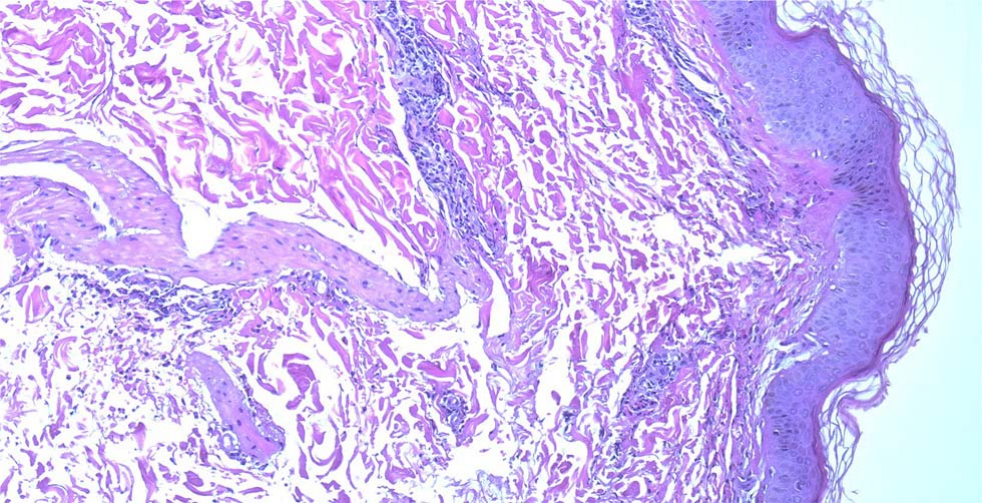
Skin biopsy showing superficial dermal small-vessel inflammation with neutrophilic infiltrate and leukocytoclasia (H&E stain, ×200).

**Figure 5:**
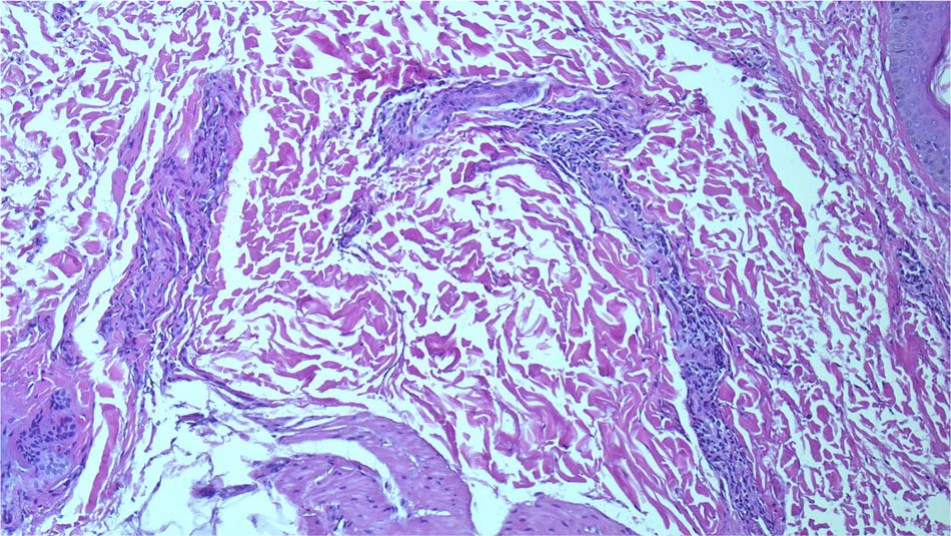
Higher-power view demonstrating nuclear dust and extravasated erythrocytes within vessel walls (H&E stain, ×400).

A skin biopsy demonstrated an unremarkable epidermis with a superficial dermal infiltrate composed predominantly of neutrophils with scattered eosinophils involving small vessel walls, associated with leukocytoclasia and extravasated erythrocytes, without fibrinoid necrosis. These findings were consistent with leukocytoclastic vasculitis (Figs 4 and 5).

At the time of presentation, the patient was clinically asymptomatic from a gastrointestinal standpoint, with persistently normal inflammatory biomarkers (low fecal calprotectin levels, low C-reactive protein, normal hemoglobin, normal platelet count), indicating sustained remission of Crohn’s disease. In the absence of clinical or biochemical evidence of active intestinal inflammation, repeat endoscopic evaluation was not pursued. Accordingly, the cutaneous vasculitic eruption was considered unlikely to represent an extraintestinal manifestation of Crohn’s disease. Infliximab was therefore discontinued, and therapy was transitioned to ustekinumab. The cutaneous lesions improved rapidly, with complete resolution within four weeks. At eight months of follow-up, the patient remains in clinical remission on ustekinumab with no recurrence of vasculitic lesions.

## Discussion

This case adds to the growing evidence that anti-TNF therapies, while highly effective in managing Crohn’s disease, may paradoxically induce immune-mediated complications such as vasculitis. LCV has been reported with several anti-TNF agents, including infliximab, adalimumab, and etanercept [7, 8]. The proposed mechanism involves immune complex deposition and complement activation in small vessels, triggering neutrophilic inflammation [3, 7].

Our patient developed vasculitic lesions after more than four years of infliximab therapy, illustrating that drug-induced LCV can arise even after prolonged disease remission. The complete resolution of skin lesions following infliximab withdrawal strongly supports a causal relationship. Similar findings have been described in other reports, where discontinuation of the culprit agent and short corticosteroid courses led to remission of vasculitis [8–10].

Importantly, systemic involvement (renal, gastrointestinal, pulmonary, or neurological) is rare but must always be excluded with appropriate laboratory and imaging investigations, including urinalysis, renal function tests, chest imaging, and in some cases endoscopic evaluation [3, 10].

The differential diagnosis of cutaneous vasculitis in Crohn’s disease is broad. First, cutaneous small-vessel vasculitis can represent an extraintestinal manifestation of IBD itself, particularly in patients with active luminal inflammation [5]. Second, infections (bacterial, viral, or fungal) must be excluded, as immunosuppressed patients are predisposed to opportunistic pathogens that can mimic vasculitic eruptions. Third, drug induced vasculitis is increasingly recognized with the widespread use of biologic and immunomodulatory agents, including anti-TNF therapies, thiopurines, and mesalamine [6, 8, 9]. In our case, the absence of intestinal activity, negative autoimmune and infectious work-up, and lesion resolution after infliximab discontinuation favored a drug-induced mechanism.

Distinguishing between inflammatory bowel disease–related vasculitis and infliximab-induced leukocytoclastic vasculitis (LCV) can be challenging. The most useful clinical indicators supporting a drug-induced etiology include a clear temporal relationship with biologic therapy, resolution following drug withdrawal, and recurrence upon rechallenge [7, 8]. In contrast, vasculitis as an extraintestinal manifestation of IBD is more commonly associated with active intestinal disease and may persist despite changes in biologic therapy [5, 9].

In the present case, the onset of leukocytoclastic vasculitis occurred in the setting of sustained remission, with normal inflammatory markers and no clinical evidence of active Crohn’s disease, strongly favoring a drug-induced mechanism rather than an extraintestinal manifestation.

Infliximab was therefore discontinued, and treatment was transitioned to ustekinumab, given its distinct mechanism of action targeting interleukin-12 and interleukin-23 and its favorable safety profile in patients who develop immune-mediated adverse events related to anti-TNF therapy. Several reports have demonstrated successful disease control with ustekinumab following antiTNF discontinuation due to autoimmune complications [11–13].

Clinicians should maintain a high index of suspicion for vasculitic eruptions in patients with inflammatory bowel disease receiving biologic therapies, even after prolonged treatment duration. Early recognition and appropriate management are essential to prevent potential systemic involvement. As the use of biologic agents continues to expand, systematic reporting of such rare adverse events remains crucial for improving pharmacovigilance and guiding clinical practice.

## Data Availability

All data relevant to this case report are included within the manuscript. No additional datasets were generated or analyzed.

## Additional Note on AI Assistance

We would also like to disclose that ChatGPT (GPT-5, 2025) was used to assist in drafting and refining the language of our manuscript. All clinical content, data accuracy, and final interpretations were reviewed and confirmed by the authors.
